# Histone deacetylase 6 interference protects mice against experimental stroke-induced brain injury via activating Nrf2/HO-1 pathway

**DOI:** 10.1080/19768354.2019.1601132

**Published:** 2019-04-18

**Authors:** Jie Li, Yanping Zhao, Junfeng Shi, Zhanyun Ren, Feng Chen, Wuzhuang Tang

**Affiliations:** Department of Neurology, Affiliated Yixing Hospital of Jiangsu University/Affiliated Yixing Clinical School of Medical School of Yangzhou University, Yixing, Jiangsu Province, Peoples’ Republic of China

**Keywords:** Histone deacetylase 6, Nrf2/HO-1, cerebral ischemia/reperfusion injury, oxidative stress

## Abstract

Cerebral stroke is a fatal disease with increasing incidence. The study was to investigate the role and mechanism of Histone deacetylase 6 (HDAC6) on experimental stroke-induced brain injury. The recombinant shRNA-HDAC6 or scramble shRNA lentivirus was transfected to ICR mice. Then, the ischemia/reperfusion injury (I/RI) mice were constructed using middle cerebral artery occlusion (MCAO) method. Brain TTC staining was used to determine infarct areas. Serum levels of oxidative stress-related factors were detected by enzyme linked immunosorbnent assay (ELISA). Realtime-qPCR (RT-qPCR) and Western blot were used to respectively detect mRNA and protein levels. HDAC6 was up-regulated in brain I/RI mice (MCAO group), and down-regulated again in MCAO mice transfected with shRNA-HDAC6 (MCAO + shRNA group). The infarct area of the MCAO mice was increased, neurological scores were higher, and serum protein levels of 3-NT, 4-HNE and 8-OHdG were higher. HDAC6 interference attenuated above effects. Though protein levels of Nrf2 and HO-1 in cytoplasm increased slightly in MCAO group, they increased significantly by HDAC6 interference. The protein levels of Nrf2 in cytoblast decreased significantly in MCAO group, and increased markedly by HDAC6 interference. HDAC6 interference protected mice against experimental stroke-induced brain injury via Nrf2/HO-1 pathway.

## Introduction

Cerebral stroke is the second most common fatal disease in the world (Wang et al. 2018). The incidence of the disease increases annually as the aged population increases (Chen et al. 2018). An average of 17 million cases of new stroke takes place each year in the world (Guo et al. 2018; Zhao et al. 2018). Ischemic stroke accounts for 60–70% of all cerebral strokes (Ismael et al. 2018; Xia et al. 2018). The treatment of conventional ischemic stroke is thrombolytic therapy, which can effectively restore the blood supply and repair damaged tissue. However, the ischemia-reperfusion (I/R) injury that occurs during thrombolysis will further aggravate the injury (Morimoto et al. 2018). A growing number of studies have demonstrated that oxidative stress, immune regulation, epigenetic modifications and inflammatory responses played important roles in ischemia-reperfusion injury (I/RI) (Deng et al. 2018; Gerzanich et al. 2018).

Nrf2 is a leucine zipper-rich transcription factor that belongs to the family of CNC transcription factors and is a central regulator of cellular defense against oxidative stress (Choi et al. 2018). Nrf2 is commonly expressed in various tissues and cells. The structure of Nrf2 contains a C-terminal leucine zipper region bZIP, which is a DNA-binding region that can bind to a small Maf protein in the nucleus to form a heterodimer, thereby recognizing and binding the GCTGAGTCA sequence on the ARE and initiating ARE regulation on Gultathione S Transferases (CSTs), Catalase (CAT), Superoxide Dismutase (SOD), Heme Oxygenase 1 (HOI), Quinine Qxidoreductase1 (NQO1) and other antioxidant enzyme gene expression (Han et al. 2017). Their increased expressions help to clear ROS, increase glutathione synthesis, reduce Quinonoids, etc., thereby protecting cells from oxidative stress and maintaining a dynamic balance of intracellular oxygen partial pressure (Li et al. 2018). Therefore, activation of Nrf2 signaling pathway is significant in the prevention and treatment of stroke.

Epigenetics draws much attention in the current research field of life science, and it plays an important role in cardiovascular diseases, tumor development and neurodegenerative diseases (Grunseich et al. 2014; Wang et al. 2016). Epigenetics mainly consists of DNA modification, non-coding RNA post-transcriptional regulation, chromatin structure remodeling, and histone modification (Liesz et al. 2013). The acetylation levels of histones and non-histones are mainly regulated by histone deacetylases (HDACs) and histone acetylases (HATs), which can change the transcription level of the target gene, and play an important role in diseases (Tsai et al. 2016). HDAC6, a member of histone deacetylase II family, was mainly expressed in brain and skeletal muscle cells, and it can regulate a variety of physiological functions (Demos-Davies et al. 2014). HDAC6 inhibition contributes to Nrf2 activation and neuroprotection (Gaisina et al. 2018). In this study, we also constructed a model of cerebral ischemia-reperfusion in mice, and studied the role and mechanism of HDAC6 on experimental stroke-induced brain injury.

As finding specific targets for treatment of I/RI is a hotspot and a major challenge in the medical field nowadays, it is of significant importance to provide reference for the treatment of cerebral stroke.

## Materials and methods

### Animals

Thirty animals used in the experiment were adult male ICR mice weighing approximately 25–28 g and were purchased from Beijing Vital River company (Beijing, China). The experimental mice were randomly divided into 3 groups (10 mice in a group) and fed in separate cages at constant temperature and humidity, and 12-hour light cycle. The procedures were conducted according to Management Regulations for Laboratory Animals and approved by the Animal Experimental Ethics Committee of Affiliated Yixing Hospital of Jiangsu University/Affiliated Yixing Clinical School of Medical School of Yangzhou University (Yixing, China). Efforts that were able to reduce animal suffering were made during the whole experimental process.

### Lentivirus transfection

The recombinant shRNA-HDAC6 and scramble shRNA lentivirus plasmid were obtained from Genepharma (Shanghai, China), and the virus titer was 1 × 10^9^ UT/mL. In this experiment, the model was prepared by intracerebral injection of lentivirus in mice using Stereotaxis instrument. Two-point injection of left cerebral cortex was employed in the experiment. A point is 1.0–2.0 mm before bregma, 3.0 mm left of midclavicular line, at the depth of 3.0 mm. B point is 0.5–1.5 mm behind bregma, 3.5 mm left of midclavicular line, at the depth of 3.5 mm. The mice were fixed on the stereotaxis instrument, the skin of head was cut and the skull was exposed. Nine microliters of scramble shRNA or shRNA-HDAC6 was injected at the rate of 0.5 µL/min, and the mice were named as NC or shRNA, respectively. Mice without any treatment were considered as control. All mice were then placed in animal rooms and carefully raised for a week before detecting HDAC6 mRNA expression and taking MCAO processing.

### Construction of I/R injury model

As model preparation, reversible middle cerebral artery occlusion (MCAO) without craniectomy was conducted (Longa et al. 1989; Deng et al. 2000; Sasaki et al. 2009) as follows: The specific steps were as follows: Cerebral ischemia in mice transfected with scramble shRNA (MCAO group) or shRNA-HDAC6 (MCAO + shRNA) was induced by MCAO under intraperitosssneal anesthesia of 3% (w/v) pentobarbital sodium (40 mg/kg). First, the common carotid artery (CCA), external carotid artery (ECA) and internal carotid artery (ICA) were fully exposed, and ECA was ligated by suture. A 0.18 mm diameter special nylon suture with spherical end (Bjsunbio, Beijing, China) was inserted into brain through ECA stump in the direction of CCA, passed ICA and stopped when occlude entered middle cerebral artery (MCA). Ultrasound Doppler flow angiometer showed that regional cerebral blood flow (rCBF) decreased 70%, suggesting successful occlusion of MCAO. Two hours after the skin was sutured, the ischemia condition was repaired by extracting the nylon suture from CCA, and the reperfusion process was established. Apart from MCAO, mice in the sham group underwent the same operation except MCAO. The indicators were analyzed 48 h after the treatment.

### Brain TTC staining and infarct size calculation

Neurological scores were assessed in accordance with the Longa’s standards 48 h after reperfusion (Longa et al. 1989). The standards were no injury as 0 grade, slight injury as 1 grade, moderate injury as 2 grade, severe injury as 3 grade, extremely injury as 4 grade. Mice were euthanized, the brain tissues were quickly removed to obtain coronal sections with a thickness of 3 mm. The slices were placed in 2% freshly configured 2,3,5-Triphenyltetrazolium chloride (TTC; Solarbio, Beijing, China) solution for 30 min's water bath at 37°C, under light-avoiding conditions. To calculate the infarct area rates, the stained slices were photographed and then analyzed by AlphaEaseFC software.

### Enzyme linked immunosorbnent assay (ELISA)

The serum levels of oxidative stress-related factors including 3-NT, 4-HNE and 8-OHdG were measured in sham, MCAO and MCAO + shRNA groups by ELISA kit (Bioswamp, China), according to the manufacturer’s instructions. The ELISA kits were Mouse 10708 Mouse 3-Nitrotyrosine (3-nt) ELISA Kit, MU30939 Mouse 4-Hydroxynonenal (4-HNE) ELISA Kit, MU30115 Mouse 8-Hydroxy-desoxyguanosine (8-OHdG) ELISA Kit respectively. Samples and standard substances were added into wells of 96-well plate and incubated at 37°C for 90 min. Biotinylated antibodies were added into the wells and then incubated for 60 min. Avidin peroxidase complex (Bioswamp, China) was added and incubated for 30 min before tetramethylbenzidine coloration. Finally, optical density (OD) values were read at 450 nm by an ArioskanTM microplate reader (ThermoFisher, USA), and samples quantities were calculated by standard curve.

### Realtime-quantitative polymerase chain reaction (RT-qPCR)

The cerebral cortex of brain tissues were obtained and sliced for RT-qPCR assay. The mRNA expression levels of HDAC6 were detected among Control, NC and shRNA groups, or sham, MCAO and MCAO + shRNA groups. Total RNA was extracted from tissues using Trizol reagent (Invitrogen, USA), and reversely transcribed to cDNA using Transcriptase (Takara, Japan). Next, the cDNA was amplified by LightCycler® Multiplex Masters (Roche, USA) in LightCycler® 480II System (Roche, USA) with HDAC6 primers as follows: forward primer: 5′-GCGCCCAGACTTCTATCTCC-3′, reverse primer: 5′-TCCAGCAATGACTTGGGCAT-3′. The process were conducted as follows: initial denaturation at 95°C for 5 min, 35 cycles (denaturation at 94°C for 30 s, annealing at 57°C for 30 s, extension at 72°C for 30 s), and final extension at 72°C for 5 min.

### Western blot

The cerebral cortex of brain tissues were obtained and sliced for protein extraction. Total proteins were extracted by using N-PER™ Neuronal Protein Extraction Reagent (Pierce, Thermo, USA). Nuclear and cytoplasmic proteins were extracted using NE-PERTM Nuclear and Cytoplasmic Protein Extraction Kit (Pierce, Thermo, USA). The protein levels of total HDAC6, HO-1 in cytoplasm, Nrf2 in cytoplasm or cytoblast were detected in sham, MCAO and MCAO + shRNA groups. Proteins were quantified by BCA protein assay kit (Pierce, USA) and subjected to 12% sodium dodecyl sulfate-polyacrylamide gel electrophoresis (SDS-PAGE) for protein separation. Separated proteins were then transferred to polyvinylidene fluoride (PVDF) membrane (ThermoFisher, USA), which was blocked using 5% non-fat dry milk at 37°C for 1 h, and probed first with specific primary antibodies overnight at 4°C, and then with secondary antibody at 37°C for 1 h. GAPDH was used for internal reference for total proteins and cytoplasmic proteins. LaminA was used for internal reference of nuclear proteins. The immunoblots were visualized by enhanced chemiluminescense (ECL) detection reagents (Pierce, USA), and analyzed by Bio-Rad ChemiDoc XRS densitometry with Image Lab™ Software (Bio-Rad, USA). The primary antibodies were from CST Company (USA): rabbit anti-HDAC6, anti-HO-1, anti-Nrf2, anti-LaminA and anti-GAPDH. The secondary antibody was anti-rabbit IgG, HRP-linked Antibody (CST, 7074, 1:5000, USA).

### Statistical analysis

SPSS 19.0 (SPSS, USA) was used to conduct statistical analysis. Data were analyzed by one-way analysis of variance (ANOVA) and Dunnett’s post hoc test. *P* < 0.05 and *P* < 0.01 represented statistical significance.

## Results

### HDAC6 was up-regulated in brain I/RI mice.

**Figure 1.**
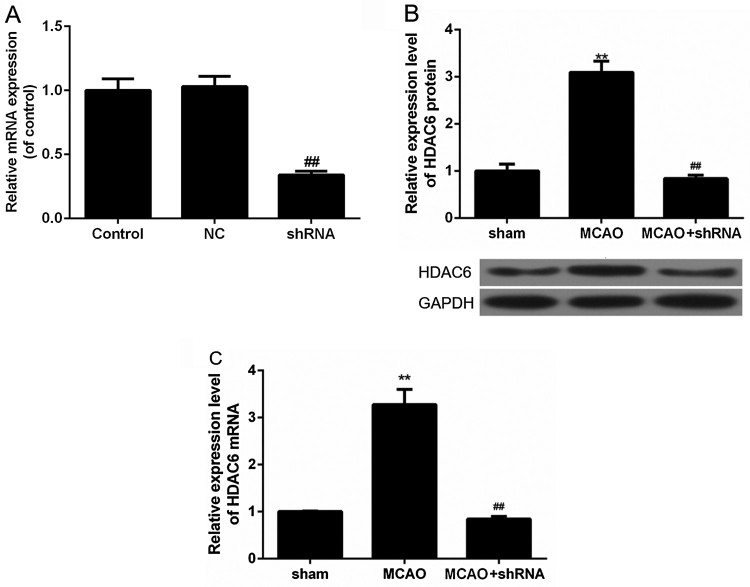
HDAC6 was up-regulated in brain I/RI mice. (A) RT-qPCR was used to analyze mRNA expression levels of HDAC6. ^##^*P *< 0.01 vs. NC group. (B) Western blot was used to analyze the protein levels of HDAC6 in brain I/R-injured MCAO group and the shRNA-HDAC6 lentivirus transfected MCAO mice (sham + shHDAC6 group). **P < 0.01 vs. sham group, ^##^*P *< 0.01 vs. MCAO group. (C) RT-qPCR was used to analyze mRNA expression levels of HDAC6. **P < 0.01 vs. sham group, ^##^*P *< 0.01 vs. MCAO group.

In order to study the role of HDAC6 in mice brain I/RI, we used the brain stereotaxic apparatus for intracerebroventricular injection of shRNA-HDAC6 lentivirus to mediate mice HDAC6 gene silencing. The mRNA expression of HDAC6 was measured one week after the injection, and the results showed that the mRNA levels of HDAC6 in shRNA group was decreased significantly, compared with NC group (Figure 1, *P *< 0.01), meaning successfully transfection. MCAO method was used for I/RI mice model construction. Western blot analysis showed that HDAC6 protein expression in brain I/R injured MCAO group significantly increased, but significantly reduced in the shRNA-HDAC6 lentivirus-transfected MCAO mice (sham + shHDAC6 group) (Figure 1(B), *P *< 0.01). Meanwhile, RT-qPCR analysis showed consistent results (Figure 1(C), *P *< 0.01), that is, HDAC6 mRNA expression was significantly increased after brain I/RI, and noticeably reduced in sham + shHDAC6 group.

### HDAC6 exacerbated brain damage and oxidative stress in brain I/RI mice.

**Figure 2.**
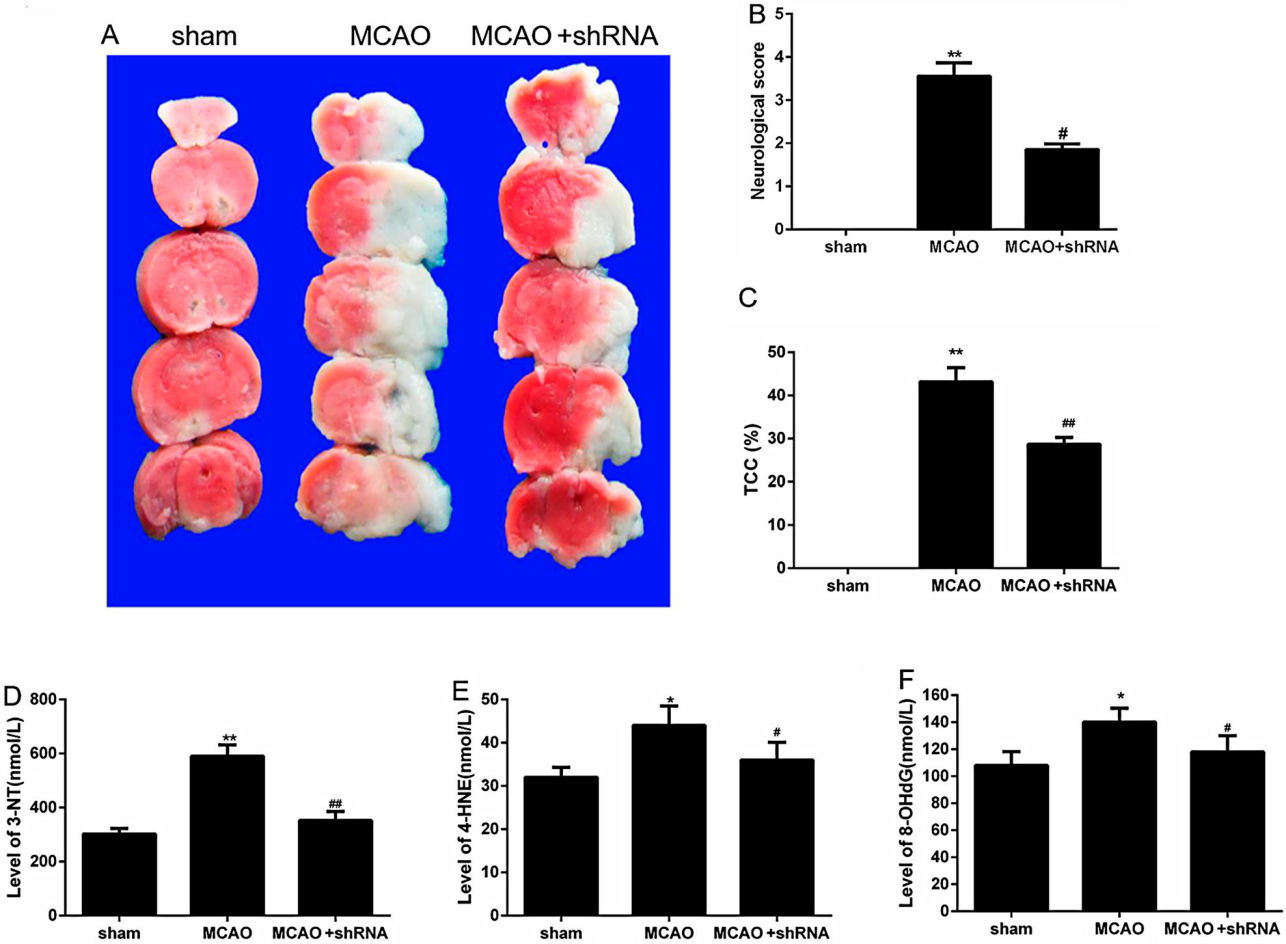
HDAC6 exacerbated brain damage and oxidative stress in brain I/RI mice. (A and C) Brain TTC staining was used to measure the cerebral infarct areas. (B) The neurological scores were compared. (D–F) The protein levels of oxidative stress-related factors including 3-NT, 4-HNE and 8-OHdG in serum were measured by ELISA. *P < 0.05 and **P < 0.01 vs. sham group, ^#^*P *< 0.05 and ^##^*P *< 0.01 vs. MCAO group.

Through TTC detection, the cerebral infarct area was found significantly increased after mice brain I/RI in MCAO group, indicating that the model was successfully constructed, and the infarct area of the MCAO mice with HDAC6 interference (MCAO + shRNA) was reduced after HDAC6 silencing (Figure 2(A, C), *P *< 0.05). This proved that HDAC6 increased the area of cerebral infarction. By neurological scoring method, we found that the neurological score in MCAO group was significantly higher than sham mice after brain I/RI, which proved that the model was successfully constructed. The neurological scores of MCAO mice with HDAC6 interference (MCAO + shRNA) were significantly lower than those in MCAO model group (Figure 2(B), *P *< 0.05), suggesting that HDAC6 aggravated brain damage. The results of ELISA showed that protein levels of oxidative stress-related factors in serum including 3-NT, 4-HNE and 8-OHdG, were up-regulated in MCAO group, but down-regulated again in MCAO + shRNA group (Figure 2(D–F), *P *< 0.05).

### HDAC6 inhibited Nrf2/HO-1 pathway in brain I/RI mice.

**Figure 3.**
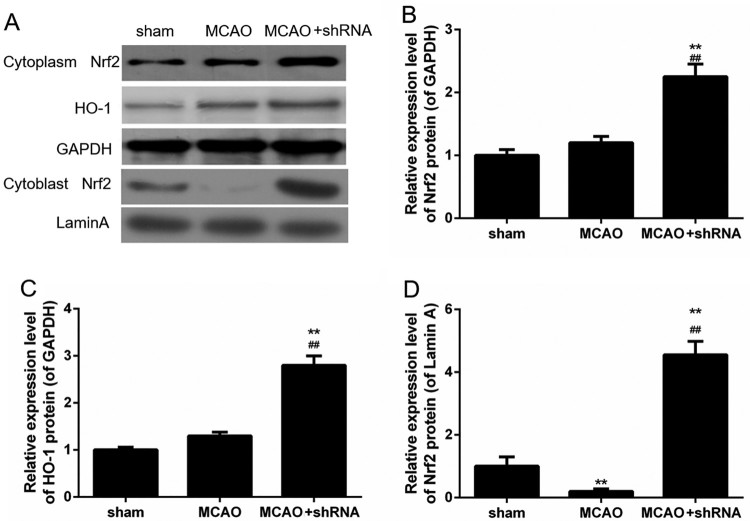
HDAC6 inhibited Nrf2/HO-1 pathway in brain I/RI mice. (A–D) The protein levels of Nrf2 and HO-1 in cytoplasm, and Nrf2 in cytoblast were determined by Western blot. **P < 0.01 vs. sham group, ^##^*P *< 0.01 vs. MCAO group.

Cytoplasmic and nuclear proteins were respectively extracted from brain tissues. Results of Western blot showed that the protein levels of Nrf2 and HO-1 in cytoplasm increased slightly in MCAO group, but significantly in the shRNA-HDAC6 lentivirus transfected MCAO mice (sham + shHDAC6 group) (Figure 3(A–C)). Such a phenomenon might be caused by slightly increased stress reaction. In addition, the protein levels of Nrf2 in cytoblast decreased noticeably in MCAO group, compared to sham group, while the protein levels of Nrf2 increased markedly in MCAO + shRNA group, compared to MCAO group (Figure 3(D), *P *< 0.01). It might indicate that HDAC6 silence promoted Nrf2 into cytoblast.

## Discussion

Cerebral stroke is a fatal disease with increasing incidence (Park et al. 2018). Oxidative stress, epigenetic modifications and inflammatory responses play important roles in I/RI (Tanaka et al. 2018). HDAC6 gene mutation is closely related to large-vessel stroke, however, the role and mechanism of HDAC6 on experimental stroke-induced brain injury still remains unclear. In our study, the classic method of MCAO was applied to construct an I/RI mice model. The expression levels of HDAC6 in MCAO mice were found markedly up-regulated, and shRNA-HDAC6 lentivirus transfected into brains of MCAO mice significantly down-regulated the expression levels of HDAC6, indicating that HDAC6 played important roles in cerebral ischemic stroke. The histological damage of injured brain regions are often associated with acute ischemic stroke. Thereafter, we examined whether shRNA-HDAC6 transfection could protect brain against functional deficits and ischemic lesion size. The neurological scores and infarct sizes were both apparently attenuated by shRNA-HDAC6 in MCAO mice. It preliminarily verified that HDAC6 silence was effective in repairing I/RI.

To our knowledge, oxidative stress is a critical factor in inducing ischemia-reperfusion injury (Vijayan and Reddy 2016). The inflammatory reaction is aggravated and the concentrations of serum inflammatory factors are activated. Therefore, it is predicted that oxidative stress factors are associated with shRNA-HDAC6 attenuating cerebral ischemia. Antioxidative enzymes are correlated to oxidative stress biomarkers of protein nitration: 3-nitrotyrosine (3-NT), lipid peroxidation: 4-hydroxynonenal (4-HNE), and 8-hydroxydeoxyguanosine (8-OHdG) (Paul et al. 2018). 4-HNE, an α,β-unsaturated aldehyde, is a lipid peroxide that caused neuronal death at high densities (Kruman and Mattson 1999). More 3-NT will be produced under oxidative stress in neurons (Gunther et al. 2018). DNA strand breaks are often caused in oxidation, and guanine transferring to 8-OHdG is frequently detected *in vivo*, as a novel biomarker for oxidation stress (Ma et al. 2013). In our study, levels of 3-NT, 4-HNE and 8-OHdG were increased significantly in MCAO mice and decreased by HDAC6 silence, indicating HDAC6 could regulate the expression of oxidative stress factors to mediate oxidative stress. However, we just detected the oxidative stress-related factors in serum, which showed good trend. It might be a limitation of the article not studying them in tissues, which would be done in future study.

To further illustrate the molecular mechanisms of HDAC6 regulating oxidative stress, Nrf2/HO-1 pathway was studied as it has a critical function on oxidative stress regulation and neural protection during I/RI process. Nrf2 and the induction of its downstream target markers, such as HO1, NQO1 and SOD were reported to be induced during the neuro-protecting process produced by Ginseng (Liu et al. 2018). The protective function of paeonol was also related to Nrf2 activation (Zhao et al. 2014). Considering the effect of Nrf2 in oxidative stress and neuro-protection, we studied the expression level of Nrf2 and its downstream target marker HO-1 under the condition of HDAC6 interference in MCAO mice. Our data showed that Nrf2 levels in cytoplasm and nuclear both increased significantly as well as levels of HO-1, showing that, HDAC6 interference not only promoted the production of Nrf2, but also promoted its movement from cytoplasm to nucleus to activate its downstream factors. Though levels of Nrf2 and HO-1 in cytoplasm were slightly higher in MCAO mice than normal mice, and such a phenomenon might be caused by slightly increased stress reaction. Together, our data showed that HDAC6 interference protected mice against oxidative stress and I/RI via activating Nrf2/HO-1 pathway.

In conclusion, the epigenetics modification played a critical function in ischemia/reperfusion injury, in which histone deacetylase HDAC6 mediated Nrf2/HO-1 pathway, and oxidative stress regulation were involved. HDAC6 silence was an effective way to attenuate I/RI by regulating oxidative stress. It might provide reference for the diagnosis and treatment of I/RI, in the field of genetics.
